# Opportunities and Challenges in Understanding Atherosclerosis by Human Biospecimen Studies

**DOI:** 10.3389/fcvm.2022.948492

**Published:** 2022-07-07

**Authors:** Maria Elishaev, Chani J. Hodonsky, Saikat Kumar B. Ghosh, Aloke V. Finn, Moritz von Scheidt, Ying Wang

**Affiliations:** ^1^Department of Pathology and Laboratory Medicine, Center for Heart Lung Innovation, University of British Columbia, Vancouver, BC, Canada; ^2^Center for Public Health Genomics, Department of Public Health Sciences, University of Virginia, Charlottesville, VA, United States; ^3^Cardiovascular Pathology Institute, Gaithersburg, MD, United States; ^4^Department of Cardiology, Deutsches Herzzentrum München, Technische Universität München, Munich, Germany; ^5^Deutsches Zentrum für Herz-Kreislauf-Forschung, Partner Site Munich Heart Alliance, Munich, Germany

**Keywords:** atherosclerosis, biobanked human biospecimens, next-generation sequencing, bioinformatic analyses, spatial biology

## Abstract

Over the last few years, new high-throughput biotechnologies and bioinformatic methods are revolutionizing our way of deep profiling tissue specimens at the molecular levels. These recent innovations provide opportunities to advance our understanding of atherosclerosis using human lesions aborted during autopsies and cardiac surgeries. Studies on human lesions have been focusing on understanding the relationship between molecules in the lesions with tissue morphology, genetic risk of atherosclerosis, and future adverse cardiovascular events. This review will highlight ways to utilize human atherosclerotic lesions in translational research by work from large cardiovascular biobanks to tissue registries. We will also discuss the opportunities and challenges of working with human atherosclerotic lesions in the era of next-generation sequencing.

## Introduction

Ischemic cardiovascular events, including heart attack and strokes, is the leading cause of mortality and morbidity worldwide (1). Atherosclerosis, the build-up of lesion cells on the wall of the blood vessels, is a chronic process underlying most ischemic cardiovascular events. Studies of human biospecimens have played an indispensable part in understanding the pathophysiology of atherosclerosis because neither cell culture nor animal models can recapitulate the complex components and structure of advanced human atherosclerotic lesions (2, 3). Before next-generation sequencing was invented, human biospecimen studies focused on the morphology and a few lesion components such as collagen, foam cells, and smooth muscle cells. Even with limited dimension, the classic histology methods have set the widely-accepted lesion classification standards and found the association between lesion morphology and ischemic events (4). The era of next-generation sequencing brought opportunities to explore the molecular features of atherosclerotic lesions in depth and *in situ*. Hence, researchers have multiple options to apply the classic and new technologies to human biospecimens. The question is how to match the research scope and approach with the human biospecimens available in large biobanks or small-scale tissue registries. This review will provide a few examples to showcase current advances in utilizing human atherosclerotic lesions, share our views of challenges in knowledge translation and vision of future needs to advance research on human biospecimens in the area of atherosclerosis.

## Current Advances in Human Biospecimen Studies

### Morphological Insights

Histology assessment is a routine in most tissue biobanks. Traditional hematoxylin-eosin and Movat's Pentachrome stainings visualize the structure and basic components of the lesions. These widely adopted methods revealed not only the association between morphology and cardiovascular events, but also risk factors that contribute to disease progression. Biopsy studies found that the morphological features of lesions underneath cardiac thrombosis fall into three categories: rupture, erosion, and calcified nodule (5), indicating the connection between morphology and risk of ischemic cardiovascular events. Following this notion, Virmani et al. developed a comprehensive assessment criteria using morphology to define the trajectory of lesion progression (5). Correlation studies can further infer the pathogenic drive of lesion progression by connecting the traditional risk factors of atherosclerosis with morphology. Burke et al. found that patients having plaque rupture also had higher cholesterol levels as recorded in their postmortem toxicological tests (6). It suggests that the traditional risk factor, cholesterol levels, drives lesion progression toward a rupture-prone structure and lipid-lowering drugs will benefit patients having this type of lesions. In addition to studying lesions at late stages, Nakashima et al. observed that vascular beds that develop diffuse intimal thickening in early childhood are also the “hot spots” for atherogenesis in later life (7). Large biobanks have a statistically powerful amount of samples to explore how lesion morphology is affected by genetic risk factors in patients of different ethnicities. CVPath Institute has the world's largest and most comprehensive collection of heart samples from more than 8,500 sudden coronary death cases. Their studies have shown a higher risk of sudden coronary death in the African American population compared to the Caucasian population (8). Guo et al. explored the genetic reasons behind this phenomenon and found that more African Americans carry a single nucleotide polymorphism rs7136716 than the Caucasian population and this genetic variant is correlated with increased expression of CD163 macrophages in ruptured lesions (9). Then they determined that CD163^+^ macrophages contribute to intraplaque microvessels and inflammation, remodeling lesion structure to be rupture-prone. This data suggests that rs7136716 is a genetic risk variant particularly enriched in patients of an African ancestry. Hence, the integration of pathology and genetics from large biobank studies can provide new insights into the discovery of biomarkers and personalized medicine.

### Molecular Phenotyping

There has been an explosive increase in atherosclerosis research using single-cell RNA sequencing (scRNA-seq). These studies have revealed the vast heterogeneity in the cell components of atherosclerotic lesions, including more than six phenotypes of SMCs (10–12) and at least three macrophage subsets (13, 14) among eleven distinct leukocyte populations (15). Most previous studies applied next-generation sequencing to animal models, in which disease stages can be controlled, and cell lineage tracing is available. These advantages made them better tools for understanding the trajectory of phenotypic changes and trans-differentiation of lesion cells in the early disease stage (10, 16). Applying next-generation sequencing to human lesions has its own technical challenges and limitations, discussed in several recent reviews (17–19). Nevertheless, single-cell sequencing of human lesions will distinguish cell phenotypes and signaling pathways in categorized patient cohorts, the differences in which can be used to predict therapeutic targets and treatment outcomes. Slenders et al. projected GWAS loci into scRNA-seq data of human carotid lesions and defined cell-specific risk genes that can be translated to therapeutic targets (20). Fernandez et al. applied several single-cell sequencing technologies to human carotid lesions. They found that differences in the interleukin-1β (IL-1β) signaling pathway between symptomatic and asymptomatic patients may lead to diverse treatment outcomes in the CANTOS trial, which aims to block IL-1β to reduce the risks of cardiovascular events (21). One limitation of using scRNA-seq to predict cellular function is that it ignores all the post-transcriptional regulation of gene expression. Quantitative proteomics uses technologies such as mass spectrometry to characterize proteins, molecules that eventually execute gene function. Since traditional mass spectrometry usually requires a few milligrams of input material, most previous studies focus on cultured cells instead of digested lesions to obtain the protein atlas of a single cell type. Okui et al. applied mass spectrometry to cultured human coronary artery SMCs and found that carnitine O-octanoyltransferase increases during osteogenic transition of SMCs, which will lead to calcification during atherogenesis (22). The Athero-Express study used proteomics to analyze the protein features of human carotid lesions and found that osteopontin was strongly associated with cardiovascular events during the follow-up post endarterectomy surgery. suggesting that pathologists can use osteopontin as a biomarker to predict patients' future risk of cardiovascular events (23). The Athero-Express study collects carotid lesions from endarterectomy surgeries and follows up with the clinical records of donors to determine the relationship between lesion composition and future adverse cardiovascular events (23). It embraces the idea that atherosclerosis is a systemic disease and biopsy tests of surgically aborted lesions will inform the progression of lesions in other vascular beds (24). The Athero-Express study has recently applied state-of-the-art scRNA-seq technology to their biobank to advance our understanding of the transcriptome landscape of human lesions (25), and more specific biomarkers are expected with accumulated data of clinical follow-up. Hence, when the molecular features of human lesions are linked to clinical follow-up of the specimen donors, it will support the discovery of biomarkers.

### Proof of Concept

Compared to large biobanks, sample availability in small-scale tissue registries is often too low to support genetic studies such as investigating the polygenic risk factors. Without follow-up of clinical outcomes, the archived tissue samples are not ideal for biomarker studies. However, these tissues can be readily used to prove the concepts of basic sciences: “reality check” that observations in animal studies or cultured cells will apply to human atherogenesis. It is now well-known that vascular smooth muscle cells (SMCs) undergo phenotypic changes during atherogenesis and most of them do not express lineage markers such as myosin heavy chain 11 and smooth muscle alpha-actin, but it was not till the invention of SMC-lineage tracing mice when researchers realized about this (26). To prove the presence of de-differentiated SMCs in human atherogenesis, Gomez et al. developed a staining method to visualize cells of SMC origin using SMC-lineage tracing mice and then applied the technique to human carotid lesions (*n* = 5) (27). Clinical information can be completely detached from the biospecimen studies in such proof of concept research and small sample size is acceptable. Working with archived samples shortens the time of ethical approval for researchers. Proof of concept studies allow archived specimens to be repurposed and maximized in the tissue registries. For example, the Cardiovascular Tissue Registry at the Centre for Heart Lung Innovation at the University of British Columbia collects hearts donated by heart transplant patients, and the myocardium has been utilized to support studies of cardiac allograft vasculopathy (28). In parallel, Allahverdian et al. characterized the atherosclerotic coronary arteries from these hearts and discovered that an underestimated amount of foam cells are derived from SMCs instead of macrophages (29). This study, along with growing evidence from GWAS of coronary artery disease (30–32), has changed the traditional dogma that atherosclerosis is mainly a macrophage-driven disease.

## Current Gaps in Knowledge Translation

Despite the current advances in new technologies, guidelines of research design (19) and customized protocols (33) to maximize the utilization of human biospecimen, translation of biobank research to biomarkers and therapeutic targets is still limited. Current gaps in knowledge translation include the lack of tailored bioinformatics tools to interpret “Omics” data, “Omics” in the tissue context, and connections of traits in the lesions with blood-borne biomarkers, which is more applicable for clinical testing.

### Bioinformatic Tools

As more human biospecimen studies have embraced scRNA-seq technology, bioinformatics tools that are primarily R- and python-based have been developed, including Seurat (34), Signac (35), archR (36), singleR (37), scCATH (38), and Garnett (39). Additionally, the less robust single-cell sequencing data can intersect with large bulk sequencing datasets, such as the Human Cell Atlas (40) through Cibersort (41) and MuSiC (42), to estimate the portion of specific cell types in the bulk-seq data. However, only a few tools incorporate established pipelines into webapps or standalone applications to allow scientists without programming experience to analyse their data. Most bioinformatics tools are also incompatible with integrating datasets published in various formats. This problem is accompanied by bioethical considerations of what clinical information affiliated with the human biospecimen can be shared and how to oversee the appropriate use of shared datasets (43–46). Furthermore, myriad analysis options are available: over 1,000 tools are developed for scRNA-seq alone (47), with limited benchmarking for atherosclerotic lesions. The nature of cells in the atherosclerotic lesion: cell plasticity and dynamic phenotypic changes during atherogenesis (10, 48), is especially challenging for bioinformatics tools that rely on: (1) reference datasets to define a cell type, and (2) the persistent presence of a phenotype in transition to trace the trajectory of atherogenic changes (49–52).

### Lack of Spatial “Omics” Data

Gene ontology analyses of scRNA-seq data predict the function of each cell subset based on signaling pathways and biological processes involving genes specific to that subset. However, cells are unevenly distributed among the atherosclerotic lesions. In the traditional diagram of a fibroatheroma, endothelial cells are located at the luminal side with SMCs underneath and macrophages form foam cells in the intima and shoulder region. After the discovery of cell subsets and phenotypic transition of lesion cells, now we know that the fibrous cap is derived from SMCs and endothelial cells that have undergone endothelial-to-mesenchymal transition (16). CD68^+^ “macrophages” in the deep intima are made of leukocytes and SMCs (29). The stability of lesion structure is affected by the distribution of these cell subsets and their interactions with the others. Within the several types of SMCs in atherosclerotic lesions, some are fibroblast-like, potentially forming a protective fibrous cap (53), whereas pro-inflammatory macrophages and SMCs may enlarge the necrotic core by altering the function of each other (54). These assumptions can be validated only when we see fibroblast-like SMCs on the fibrous cap of stable lesions and more impaired macrophages are close to the pro-inflammatory SMCs near the necrotic core. In the absence of lesion context, it can be misleading to predict lesion progression purely based on signaling pathways and biological processes represented by gene expression. The same mitogenic signaling pathway turned on by IL-1β in SMCs may cause pathogenic expansion of the atherosclerotic lesions (55) when these SMCs are located in the intima, but it may be athero-protective when these cells are in the fibrous cap. Blocking IL-1β in animals with established fibroatheroma resulted in lesion destabilization, suggesting that at least a subset of SMCs executed IL-1β's mitogenic effects in an athero-protective manner by investing into the fibrous cap (56). Hence, it is critical to consider spatial “Omics” data when translating single-cell “Omics” data to therapeutic targets and biomarkers.

### Connection to Traits in the Blood

Tissue biopsy examinations are well-accepted for phenotyping tumors but are not practical in the clinical practice of atherosclerotic disease where not all patients require surgical treatment. Connecting the traits of atherosclerotic lesions to those in the blood will help the discovery of blood-borne biomarkers that can be easily implemented in clinical laboratories. Phenotyping blood cells has the potential to reveal cellular biomarkers in the circulation (57). Hamers et al. explored protein expression of circulating monocytes using a 39-plex CyTOF panel and found a positive correlation between Slan^+^CXCR6^+^ monocyte population in the blood and the severity of atherosclerotic disease estimated by medical imaging, suggesting that this monocyte population could be a biomarker for disease progression (57). More importantly, studying biopsy and blood samples from the same donor will determine whether molecules in the blood mirror biological activities in the lesion. The interleukin-6 (IL-6) signaling pathway is a crucial mediator of inflammation in the lesions (58), and elevated plasma IL-6 in patients with acute coronary syndrome is currently the most powerful predictor of increased mortality in both short and long terms (59, 60). To explore its utilization as a biomarker for risk of cardiovascular events, the Biobank of Karolinska Endarterectomies study found that plasma IL-6 is positively correlated with those in the carotid lesions (61), but no significant differences were observed between symptomatic and asymptomatic patients (62). Hence, plasma IL-6 does not precisely reflect lesion progression and risk of adverse cardiovascular events. Until now, we have not found clinically applicable biomarkers to mirror biological activities in atherosclerotic lesions. Clonal hematopoiesis of indeterminate potential (CHIP), a phenomenon by which blood precursor cells in bone marrow obtain mutations during aging, is associated with a higher risk of atherosclerosis (63). In this original study, the authors found more than one hundred CHIP mutations in peripheral blood cells but did not have access to any molecular information about the atherosclerotic lesions. This study left the knowledge gap on how specific CHIP mutations will reflect lesion progression and can be promising biomarkers for risk prediction.

## Opportunities and Challenges in Future Studies

Collaborations are the key to advancing knowledge translation of human biospecimen studies. Publicly available “Omics” data from multiple studies can be pooled together to enhance the diversity of the studied population given that most data are based on European descendants. For researchers who already have the datasets, meta-analysis will combine different studies, as applied in the Coronary Artery Disease Genome-wide Replication and Meta-analysis plus The Coronary Artery Disease Genetics consortium. It is critical for researchers working on human atherosclerotic lesions to assess the potential pitfalls of applying a bioinformatic pipeline tested on other tissues to work on atherosclerotic lesions, which requires communication between the dry and wet labs to reflect on the principles of the pipelines and biological features of the tissues. For example, a spectrum of early to late staged atherosclerotic lesions need to be incorporated into the research design to answer questions about cell trajectories. For bioinformaticians, a consortium that integrates RNA-seq datasets has not been established yet, but initiatives have been made for sharing datasets in a user-friendly way. PlaqView is the first platform that empowers researchers who do not have access to human atherosclerotic lesions or who do not have bioinformatics expertise to re-analyze published scRNA-seq data (64). More biobanks have started to investigate blood and lesion samples from the same donor side-by-side to develop blood-borne biomarkers. The recently established Munich Cardiovascular Studies Biobank at the German Heart Center Munich has collected more than 800 pairs of blood and lesion samples to connect molecular traits in atherosclerotic lesions to those in blood. Small-scale tissue registries have the opportunity to collaborate with biobanks that have access to blood samples and patients' clinical follow-up records to validate molecular targets or biomarkers found in tissue studies. Spatial gene expression (Table 1) and multiplex imaging technologies (Table 2) have been commercialized to optimize the use of archived tissue blocks by measuring gene and protein expression in a high-throughput fashion. Roadblocks to applying them to human atherosclerotic lesions are mainly technical. Regarding histology integrity, lesion sections can easily fold and tear during sample preparation due to the natural curvature of the lumen and the presence of calcification and large necrotic cores. Regarding quality control of the RNA, atherosclerotic lesions, especially the highly necrotic ones, have low cellularity compared to the more frequently reported tumor, brain, and myocardium tissues. Based on our experience and previous research (65), a large part of one lesion (at least 100 μm long) is required to extract an adequate amount of RNA for quality assessment: 5 ng for the 2100 Agilent bioanalyzer (RNA 6000 Nano kit) or RT-PCR to amplify housekeeping genes. Moreover, Formalin-Fixed Paraffin-Embedded is the standard archiving format in biobanks to preserve lesion morphology. RNA stability remains unchanged for up to 10 years in this format (65). Previous research found that RNA is unevenly degraded within the same lesion section (66). Hence, we think that *in situ* assessment of RNA quality (67) is more suitable than extracting RNA from the whole section to select samples for spatial gene expression. Such a method can advise which lesion region has high quality input material to generate sequencing reads of high fidelity. Regarding the interpretation of data, cell segmentation has been the most problematic step for all the spatial biology tools. So far, most software and analysis pipelines assign the signals to a cell nucleus nearby based on algorithms trained in cancer tissues (68–70), assuming that cells in other tissues have similar size and shape. This may lead to inaccurate cell segmentation in lesion sections when the spindle-shaped 200 μm long SMCs, spherical lymphocytes of 7–10 μm in diameter, and foam cells with various sizes are all in close proximity. For spatial gene expression, transcriptomes from multiple cells can be mixed in one captured region and devolution of cell types requires reference scRNA-seq data (71, 72). Unlike working with dissociated cells, multiple sections are required to assess the entire lesion using spatial biology technologies, significantly increasing the cost. Removing these roadblocks requires efforts from both the academic and the technology industries.

**Table 1:** Spatial gene expression technologies.

**Technology**	**Resolution**	**Applications**	**Time**	**Common features/differences**
*Visium* Tissue sections are mounted on top of capture spots. Each spot has a unique spatial barcode to retrieve its location. For frozen tissues, mRNA is directly released to bind to spatially barcoded oligonucleotides on the capture spots. For FFPE tissues, mRNA is first hybridized with pairs of specific probes for each targeted gene. Probe pairs are then ligated and released to bind to the spatial barcode on the capture spot (73)	Capture spot of 55 μm diameter; 100 μm distance between the centers of two capture spots (73) Maximum capture area: 6.5 × 6.5 mm^2^ and 4,992 spots per capture area (73)	Frozen: Heart (74–76), Liver (77), Spinal cord (78), Skin (79), and Breast cancer (80) FFPE: Brain, ovarian cancer, lung, and kidney (81)	Hours to days (82)	- Fixed positions of capture spots include 0 to 10 cells. - RNA capture utilizes pre-existing lab equipment. - Gene expression data is layered over a morphological image of the same tissue section. - 4–5 protein markers can be combined by immunofluorescence staining with gene expression.
*GeoMx DSP* Tissue sections are stained with a mix of RNA or antibody probes, each contains a unique UV-cleavable oligonucleotide barcode. These barcodes are released by UV light illuminated at defined regions of interest (ROIs) and then counted. Reads are mapped back to each ROI, generating a map of genes or/and proteins expression within the tissue architecture (83)	As low as 10 μm (73) Minimum ROI: 5 × 5μm^2^ Maximum ROI: 660 × 785 μm^2^ (84)	FFPE*: Transcriptome*: Lymphoid and colorectal (83), Kidney (85), Brain tumor (86), Heart, lung, and liver (87) *Proteins:* Breast cancer 70-plex (88)	10–20 tissue sections in 1.5–2.5 days (depends on the number of ROI) (83)	- Flexible choice of regions respects boundaries of cells and tissue components. - RNA/protein capture require a specific instrument. - Use fluorescent morphology markers to guide selection of ROIs. - Up to 96 protein markers can be counted by nCounter, or more than 100 proteins using next-generation sequencing

**Table 2:** Spatial protein expression technologies.

**Technology**	**Resolution**	**Applications**	**Time**	**Common features/differences**
*PhenoCycler (CODEX)* Tissue sections are stained with a cocktail of antibodies conjugated with unique oligonucleotide barcodes. For each imaging cycle, reporters that carry fluorescence dyes and oligonucleotides complementary to the barcodes will bind to the antibodies to visualize the locations of 3 targeted proteins. These reporters are then removed and the cycle will repeat until all proteins are imaged.	200 nm (89, 90) Single cell level Maximum addressable sample size depends on the microscope and objective lens	FFPE: Colorectal cancer 56-plex (91) and Bladder cancer 35-plex (92) Frozen: Muscle 9-plex (93)and Spleen 30-plex (94)	Depends on the number of probes. 30-plex in 3.5 h (93)	- Autofluorescence from tissue exists. - Signals from three antibodies are captured per cycle. - Number of antibodies is theoretically unlimited. - Capable of imaging large tissue section. - Tissue section reusable
*Hyperion (IMC)* Tissue sections are stained with a cocktail of antibodies conjugated with metal tags. Stained tissue sections are ablated by a laser beam focused at 1 μm and then nebulized. The ionized metal tags are distinguished by the differences in the time of flight in the mass spectrometry.	1 μm^2^ (73, 89) Single cell level Maximum size: 15 × 55 mm^2^; Maximum ROI: 2.25 mm^2^ (95)	FFPE: Oropharyngeal cancer 33-plex (96), Lung cancer 14-plex (97), Bladder cancer 34-plex (98), and Brain 11-plex (99)	2 h to scan 1.5 mm^2^ using 200 spots/sec speed (90)	- Not affected by tissue autofluorescence. - Signals from all the antibodies are captured simultaneously. - Number of antibodies limited by available metal isotopes (~40). - Tissue section not reusable
*MIBIscope* Tissue sections are stained with a cocktail of antibodies coupled to metal tags. The primary ion beam strikes the samples to liberate the lanthanide adducts of the bound antibodies. This generates the second ions that are analyzed by the mass spectrometer. MIBI utilizes adjustable ion beams to accommodate sample acquisition at varying depth and spot size.	200 nm to 1 μm (89, 100) 5–30 nm (101) Single cell and subcellular levels Range of ROI: 400 × 400–800 × 800 μm^2^ (100)	FFPE: Breast cancer 37-plex (102), Tuberculosis lung 37-plex (103), Lymphoid, bladder, and placenta 16-plex (104)	25 min for two fields of 80 μm diameter (105) 90 ROIs (800 × 800 μm^2^ each) per day (100) 1 mm/5 h with 500 nm resolution (90)	- Similar to Hyperion but resolution can reach subcellular level

## Conclusion

Human atherosclerotic lesions carry valuable morphological and molecular information to decipher the mechanism of atherogenesis and reveal therapeutic targets and biomarkers for patients. While large biobanks with adequate sample numbers and clinical data can perform genetic and biomarker studies, a smaller sample pool in the tissue registry also plays a vital role in translating basic sciences to a human disease scenario. New biotechnologies, along with bioinformatic tools to process the data, are modernizing biobank-based research in atherosclerosis, providing opportunities to maximize the utilization of human biospecimen. However, these new tools validated in other tissues are not one-size-fits-all, given the complex cell components in human atherosclerotic lesions. Collaborations from researchers in cardiovascular disease, bioinformaticians, and technology developers are essential for benchmarking and customization of available tools to address the unique challenges in future studies of human atherosclerotic lesions.

## Author Contributions

YW designed the topic of this review article and wrote 70% of the manuscript with ME. ME performed literature review and designed the tables. SG, CH, AF, and MS wrote the rest of the manuscript and contributed to the design of review topic. All authors contributed to the article and approved the submitted version.

## Funding

This study was supported by the Canadian Institutes of Health Research (GR022678 to YW) and the Fondation Leducq (PlaqOmics 18CVD02 to AF and its Junior Investigator Award to CH, SG, MS, and YW).

## Conflict of Interest

The authors declare that the research was conducted in the absence of any commercial or financial relationships that could be construed as a potential conflict of interest.

## Publisher's Note

All claims expressed in this article are solely those of the authors and do not necessarily represent those of their affiliated organizations, or those of the publisher, the editors and the reviewers. Any product that may be evaluated in this article, or claim that may be made by its manufacturer, is not guaranteed or endorsed by the publisher.
